# Proteomic Profiling of Non-Muscle Invasive Bladder Cancer Reveals Stage-Specific Molecular Signatures and Prognostic Biomarkers

**DOI:** 10.3390/proteomes13040065

**Published:** 2025-12-10

**Authors:** Lorenza Vantaggiato, Marco Frisenda, Enxhi Shaba, Chiara Splendore, Beatrice Sciarra, Luca Bini, Alessandro Sciarra, Claudia Landi

**Affiliations:** 1 Functional Proteomics Laboratory, Department of Life Sciences, University of Siena, 53100 Siena, Italy; lorenz.vantaggiato2@unisi.it (L.V.); enxhi.shaba@unisi.it (E.S.); luca.bini@unisi.it (L.B.); 2 Department Materno Infantile e Scienze Urologiche, University Sapienza, 00185 Rome, Italy; marco.frisenda@uniroma1.it (M.F.); chiara.splendore@uniroma1.it (C.S.); alessandro.sciarra@uniroma1.it (A.S.); 3 Chemistry Department, Sapienza University, 00185 Rome, Italy; beatrice.sciarra@uniroma1.it

**Keywords:** NMIBC, pTa-LG, pT1-HG, proteomics, prognostic markers, PDC6I/ALIX, 14-3-3ε, SOD2

## Abstract

**Background**: Non-muscle invasive bladder cancer (NMIBC) comprises high-grade (HG) and low-grade (LG) variants, classified by aggressiveness, recurrence risk, and stage—either non-invasive (pTa) or invading the lamina propria (pT1). Cystoscopy remains the diagnostic gold standard, with no less-invasive alternatives, while molecular mechanisms driving tumorigenesis and treatment response are poorly understood. **Methods**: To address this gap, we conducted a preliminary top-down proteomic study on fresh biopsies from pTa-LG and pT1-HG NMIBC at initial diagnosis to identify molecular differences and potential prognostic biomarkers. **Results:** Distinct protein profiles were observed between stages. Highly abundant proteins in pT1-HG were associated with nitric oxide biosynthesis, signal transduction, inhibition of apoptosis, protein folding, and immune response. Proteins of low abundance were related to cellular localization, cytoskeleton organization, cell adhesion, phagocytosis, and tissue development. Notably, multiple proteoforms of PDC6I/ALIX, a protein implicated in the regulation of apoptosis, proliferation, and PD-L1 surface presentation, were significantly downregulated in pT1-HG tumors. Furthermore, the abundance of proteins such as GANAB, GALE, THIC, SEPT8, and MYDGF/C19orf10 correlated with tumor size, suggesting their potential as prognostic biomarkers. **Conclusions**: These proteins, taken together, indicate that they may serve as valuable prognostic markers, offering a path toward more personalized management of NMIBC beyond the traditional one-size-fits-all approach.

## 1. Introduction

Bladder carcinoma (BC) is among the ten most frequent tumors in humans worldwide, accounting for more than 90% of urothelial tumors [1]. The main risk factors are advanced age (70–84 years) associated with exposure to tobacco smoke, aromatic amines, and benzene, due to age-related reduced DNA repair capacity [2]. BC can be further classified based on its invasiveness as non-muscle invasive bladder cancer (NMIBC) or muscle-invasive bladder cancer (MIBC), a categorization closely related to the “Tumor, Node, Metastasis” (TNM) classification. Mucosal tumors (pTa and pTis) and tumors that have invaded the lamina propria (pT1) are NMIBC. In contrast, tumors that have reached the bladder wall muscle (T2), perivesical fat (T3), or nearby organs (T4) are grouped as MIBC. Another tumor classification is based on the degree of malignancy, as determined by the anatomical pathologist, which reflects the growth rate and aggressiveness of the neoplasm. Low-grade (LG) tumors are characterized by well-differentiated cytoarchitecture, indicating slower growth and lower aggression. In contrast, high-grade (HG) tumors grow more rapidly and have greater potential to spread. They exhibit disrupted cellular architecture and poorly differentiated cells, reflecting their higher malignancy. Approximately 75% of patients present NMIBC stages pTa and pT1; an accurate grading of these tumors is of clinical importance due to their association with increasing recurrence and progression rates [3]. Currently, cystoscopy is the gold standard method for the diagnosis of bladder cancer, an invasive procedure that causes discomfort to the patient and, in some cases, complications such as haematuria, urinary tract infection, or lesions of the urethra and bladder mucosa [4]. The European Organization for Research and Treatment of Cancer (EORTC) and the scoring system from the Spanish Urological Club for Oncological Treatment (CUETO) for NMIBC classify patients into low, intermediate, or high risk for recurrence and progression to muscle invasion based on factors including grade, stage, tumor size, multifocality, variant histology, lymphovascular invasion, and prior therapy [4], but current procedures for bladder cancer diagnosis suffer from a lack of clinical sensitivity and specific screening tests [5]. Evidence shows that recurrence rates in the early stages of bladder cancer are high and treatments are inadequate for preventing advanced stages [5]. For NMIBC, Bacillus Calmette–Guerin (BCG) is the gold standard treatment for high-grade and high-risk NMIBC to reduce or prevent both recurrence and progression after initial transurethral resection of a bladder tumor (TURBT) [6]. BCG is formulated as a lyophilised powder that is reconstituted in sterile saline solution before intravesical instillation. Derived from attenuated Mycobacterium bovis, BCG stimulates a robust macrophage-mediated immune response, primarily through the presentation of BCG surface antigens [7]. Therefore, biomarkers have promising values to predict pathological conditions and can be considered as effective targets for early diagnosis, prognosis, and anti-tumor immunotherapy. The biomarker discovery requires an upstream molecular characterization of the tumor subtypes, which can certainly aid in patient management with specific therapy. To this purpose, numerous genomic and transcriptomic analyses were performed on MIBC subtypes and bladder cancer treated by different therapies [8,9,10,11] while NMIBC remains less investigated. Several studies reported common mutations in LG NMIBC that predominantly affect genes of the FGFR3, PIK3CA, STAG2, and RTK/RAS/RAF pathways, while in HG neoplasia/advanced or MIBC, the mostly mutated genes are ERBB2, p53, RB1, MDM2, CDKN2A, KDM6A, and ARID1A [4]. Numerous diagnostic tests were developed and validated by the Food and Drug Administration (FDA) and European Medicines Agency (EMA) in recent years, such as the bladder tumor antigen (BTA) [12], nuclear matrix protein 22 (NMP22) [13], ImmunoCyt/uCyt+, and UroVysion as urinary biomarkers for the detection and surveillance of urothelial carcinomas, as reported by Soria F et al. [14]. However, these tests alone cannot substitute cystoscopy, given their loss of clinical sensitivity and specificity [5]. The clinical management of urothelial bladder cancer remains a significant challenge and requires comprehensive multi-omics analysis to achieve better disease stratification. Identifying at-risk patients requires identifying the molecular profiles that best predict disease course. Understanding the molecular alterations associated with disease progression and response to therapy could improve routine diagnostic and therapeutic procedures [15]. The application of proteomic studies for the identification, quantification, and profiling of proteins for NMIBC in different histological subtypes could delineate a panel of biomarkers defined as proteomic patterns achieving higher sensitivity and specificity than single proteins, in the diagnosis and prognosis of bladder cancer. For this purpose, we propose a preliminary proteomic comparison between pTa-LG and pT1-HG NMIBC biopsies obtained from patients with a first diagnosis of urothelial carcinoma of the bladder suspected by ultrasound, urinary cytology, and cystoscopy, and with histological confirmation after TURBT. The analysis revealed a characteristic protein pattern distinguishing pTa-LG from pT1-HG biopsies, highlighting proteins correlated with tumor size with a potential prognostic value.

## 2. Materials and Methods

### 2.1. Population

NMIBC tumor biopsies were collected and provided by Professor Alessandro Sciarra’s research group from the Department of Maternal and Child Health and Urological Sciences at Sapienza University of Rome, Italy. The population consisted of 11 biopsies diagnosed as pTa-LG and 11 as pT1-HG NMIBC. Patients aged between 18 and 85 years of any ethnicity with a first diagnosis of urothelial carcinoma of the bladder suspected based on ultrasound, urine cytology, and cystoscopy, and histologically confirmed after endoscopic resection of the lesion (TURB), were included in the study. Patients with a history of TURB procedures, prior local or systemic therapies that may have affected tumor cell growth (such as chemotherapy or immunotherapy), other active malignancies or those currently under treatment, ongoing oncologic therapies (including chemotherapy or targeted therapies), current hormonal or steroid treatments, or medications known to interfere with the study assessments, as well as those with a histological diagnosis of muscle-invasive bladder carcinoma, were excluded from the study. Patients’ information, such as age, weight (kg), BMI, diabetes %, smoker %, hypertension %, haematuria %, and tumor size (cm), was taken into consideration. All patients gave their written informed consent to the study, which was approved by the ethics committee of the University of Rome “La Sapienza”—Umberto I Polyclinic Hospital on 24 May 2023.

### 2.2. Analysis Workflow

**Figure 1:**
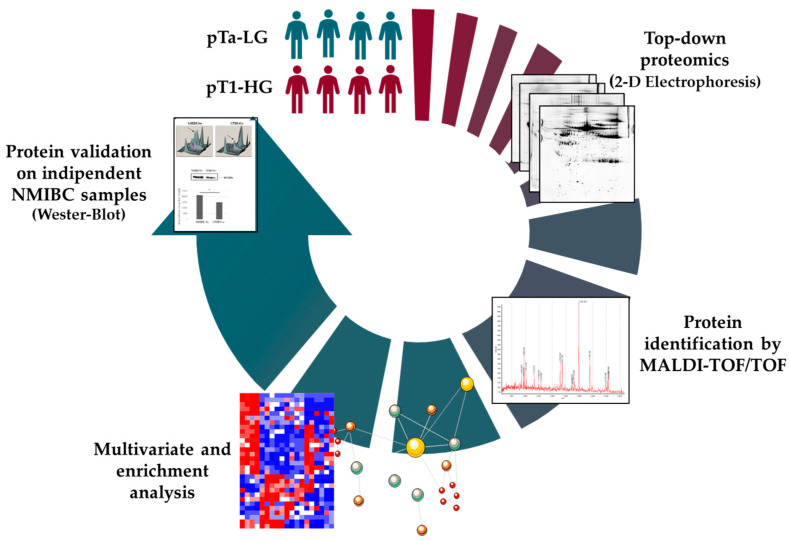
Experimental workflow.

NMIBC subtypes were chosen given the early stage of the tumor. We performed proteomic analysis on 4 pTa-LG and 4 pT1-HG subtypes since their quite different tumor stage indicates a different severity and requires a different therapeutic approach. Biopsies were selected given similar age and sex. After protein extraction and denaturation, samples were resolved by two-dimensional electrophoresis (2DE). Comparison of the 2D-gel images by bioinformatic analysis highlighted differential protein spots identified by MALDI-ToF mass spectrometry (MS). Differential proteins were used to perform multivariate statistical analysis and enrichment analysis. Protein abundance correlation with the tumor size was also performed. Based on all this data, some interesting proteins have been taken into consideration as potential prognostic biomarkers for further validation by Western blot. The experimental workflow is reported in Figure 1.

### 2.3. Sample Preparation for Proteomic Analysis

Eleven pTa-LG and eleven pT1-HG NMIBC biopsies were provided for the preliminary proteomic analysis and Western blot validation to highlight the most relevant differences between these two NMIBC subtypes. Samples were denatured with a buffer consisting of 7M Urea, 2M Thiourea, 4% (*w*/*v*) 3-[(3-Cholamidopropyl)dimethylammonium]-1-propane sulfonate (CHAPS), 1% (*w*/*v*) 1,4-Dithioerythritol (DTE), and 0.1% (*v*/*v*) TritonX100, and homogenized on ice by sonication (Ultrasonic Processor, Fisher Brand; Waltham, MA, USA; 120 watt and 20 kHz) for 2 cycles of 10 s each for a total of 40 s. Subsequently, the homogenized samples were vigorously shaken and centrifuged at 20,000× *g* for 15 min at 4 °C. The pellet was discarded, and the supernatant was used to determine the protein concentration by the Bradford method [16].

### 2.4. Two-Dimensional Electrophoresis (2DE)

Eight NMIBC biopsy samples—four pTa-LG and four pT1-HG—were processed as previously described and analyzed by two-dimensional electrophoresis (2DE) to separate intact proteins according to their isoelectric point (pI) and molecular weight (MW) prior to identification, allowing for the distinction of proteoforms. Analytical and preparative electrophoretic runs for subsequent mass spectrometry (MS) analysis were performed by loading 60 µg and 600 µg of total protein, respectively, supplemented with 0.2% and 2% carrier ampholytes. Protein loading was carried out by passive rehydration onto 18 cm immobilized nonlinear pH 3–10 gradient IPG strips (Cytiva, Uppsala, Sweden; formerly GE Healthcare). Isoelectric focusing (first dimension) was performed using an Ettan™ IPGphor™ system (GE Healthcare, Uppsala, Sweden) at 16 °C under the following voltage program: 200 V for 8 h, ramped from 200 V to 3500 V over 2 h, held at 3500 V for 2 h, increased from 3500 V to 5000 V over 2 h, held at 5000 V for 3 h, ramped to 8000 V over 1 h, and maintained at 8000 V until reaching a total of 90,000 VhT. After focusing, strips were rinsed with deionized water and equilibrated sequentially for 12 min in a buffer containing 6 M urea, 2% *w*/*v* SDS, 2% *w*/*v* DTE, 30% *v*/*v* glycerol, and 0.05 M Tris-HCl (pH 6.8), followed by a 5 min incubation in a similar buffer where DTE was replaced with 2.5% *w*/*v* iodoacetamide, containing a trace of bromophenol blue. The second dimension was carried out at a constant current of 40 mA per gel on 9–16% SDS–polyacrylamide linear gradient gels (18 × 20 cm, 1.5 mm thick) at 9 °C. Gels were visualized with ammoniacal silver staining, whereas those intended for MS analysis were stained using an MS-compatible silver staining protocol [17]. Two-dimensional gel images were acquired using an Image Scanner III laser densitometer equipped with LabScan 6.0 software (GE Healthcare), and image visualization and comparative analysis were performed with Melanie 9 software (Geneva Bioinformatics–GeneBio, Geneva, Switzerland).

### 2.5. Protein Identification by Mass Spectrometry MALDI-ToF

Protein spots showing differential abundance were manually excised from the MS-preparative 2D gels and de-stained using a freshly prepared solution containing 30 mM potassium ferricyanide and 100 mM sodium thiosulfate. The gel pieces were then rinsed six times with deionized water, followed by incubation with 200 mM ammonium bicarbonate (AB) for 30 min. After an additional wash in deionized water, the spots were dehydrated with 100% acetonitrile (ACN) and kept in this state until mass spectrometry (MS) processing. Prior to digestion, the gel fragments were rehydrated in a solution of sequencing-grade trypsin (5–10 ng/mL) prepared in 50 mM ammonium bicarbonate and incubated overnight (approximately 16 h) at 37 °C. Subsequently, 1 µL of the resulting peptide solution was applied onto a MALDI target plate, air-dried, and overlaid with a matrix solution containing 5 mg/mL α-cyano-4-hydroxycinnamic acid (CHCA) dissolved in 50% (*v*/*v*) ACN and 5% (*v*/*v*) trifluoroacetic acid (TFA), and then dried again before analysis. Mass spectrometric analyses were carried out on an UltrafleXtreme™ MALDI-ToF/ToF mass spectrometer (Bruker Daltonics, Bremen, Germany) equipped with a 200 Hz smartbeam™ I laser and operated in positive reflector mode. Instrumental parameters were as follows: 80 ns delay; ion source 1 voltage, 25 kV; ion source 2 voltage, 21.75 kV; lens voltage, 9.50 kV; reflector 1 voltage, 26.30 kV; and reflector 2 voltage, 14.00 kV. The laser operated at a wavelength of 353 nm and a frequency of 100 Hz, with the laser power set to 60%. Each spectrum represented the average of 1500 laser shots acquired from five different positions on the target spot. Spectra were automatically collected and processed using FlexAnalysis software version 3.0 (Bruker Daltonics, Bremen, Germany), which generated peak lists based on a signal-to-noise ratio threshold of 3, retaining up to 200 peaks. Internal calibration was performed using peptides derived from trypsin autoproteolysis. The mass lists were subsequently filtered to remove contaminant peaks, including matrix-related signals, trypsin autolysis fragments, and keratin peptides. Peptide Mass Fingerprinting (PMF) was carried out using the MASCOT search engine (Matrix Science Ltd., London, UK; http://www.matrixscience.com; 27 January 2025). Searches were restricted to Homo sapien entries in the Swiss-Prot/TrEMBL databases, applying the following parameters: precursor ion mass tolerance of 20 ppm, one permitted missed cleavage, carbamidomethylation of cysteine as a fixed modification, and oxidation of methionine as a variable modification. The mass spectrometry proteomics data, such as the spectrum obtained from the spectrometer and the results obtained by processing the mass list on MASCOT, have been deposited to the ProteomeXchange Consortium via the PRIDE [18] partner repository with the dataset identifier PXD069436. Each file is named with the spot number it refers to. Furthermore, the results are summarized in Table 2.

### 2.6. Statistical Analysis

Differential proteomic analysis was performed comparing the percentage of relative volume (%V) of the 2DE protein spots among the groups (pTa-LG vs. pT1-HG). The ANOVA test was applied to obtain a statistically valid *p*-value (*p*-value ≤ 0.05), and protein spots with a fold change of at least 1.5 in the %V means ratio were considered differentially abundant.

Multivariate statistical analysis was performed, taking into consideration the percentage of relative volume (%V) of the differentially abundant proteins found. Principal component and heatmap analyses were performed by XLStat software (XLSTAT-life Science—Paris, France; 2019; date: 11 July 2025). Principal component analysis (PCA), based on Pearson’s correlation, simplifies the amount of data (%V) by linear transformation, visualizing the samples into a two-dimensional plane based on the differential spot patterns. The protein spots’ clustering by heatmap was performed by using Ward’s clustering method and Euclidean distance, visualizing protein spot abundance in the rows and samples in the columns. Pearson’s correlation coefficients were applied to determine the linear correlations between %V of the differential proteins and the tumor size to determine the linear relationship by using XLStat software.

### 2.7. Enrichment Analysis

The accession numbers (AC) of the identified proteins underwent functional analysis by the DAVID Bioinformatic Resources (6.8) online tool (https://davidbioinformatics.nih.gov/; date 25 August 2025) in order to delve deeper into the biological meaning behind the list of proteins. A functional annotation tool was chosen, and our accession numbers were uploaded, selecting “uniprot_accession” as identifier and “gene list” as list type. Gene ontologies were selected among the annotation summary results.

Enrichment analysis was also performed, submitting the UniProt accession number of the identified proteins to the MetaCore™ version 22.1 building tool (Clarivate Analytics, Philadelphia, PA, USA; date 3 July 2025). MetaCore has a manually annotated database of protein interactions and metabolic reactions from the scientific literature. Using different tools, we extrapolated different information through an enrichment process. “Built network and Process network” were the selected tools for the analysis of our differential proteins.

### 2.8. Western Blot Analysis

For mono-dimensional Western blot (1DWB), 40 μg of proteins from 7 pTa-LG and 7 pT1-HG NMIBC biopsies, independent from samples used for proteomics, were combined with Laemmli buffer: 2% (*w*/*v*) SDS, 100 mM Tris–HCl pH 6.8, 4% (*v*/*v*) β-mercaptoethanol, and 20% (*v*/*v*) glycerol, and heated at 95 °C for 5 min. Samples were then resolved on a 10% polyacrylamide gel and transferred onto a nitrocellulose membrane (Hybond ECL, Cytiva, Uppsala, Sweden) as previously described by Towbin [19]. Reversible Ponceau Red staining (0.2% *w*/*v* Ponceau S in 3% *v*/*v* trichloroacetic acid) was applied to check the correct protein transfer and equal protein loading. Information about the antibodies used is reported in Table S1. Nitrocellulose membranes were washed 3 times in blotting solution: 3% (*p*/*v*) skimmed milk and 0.1% (*v*/*v*) triton X100 in Phosphate-Buffered Saline (PBS), pH 7.4, and they were incubated ON at 4 °C with primary antibodies against ALIX, SOD2, 14-3-3ε, and GANAB. After 3 washing stations in blotting solution, membranes were incubated for 2 h with secondary antibodies (Table S1). Membranes were washed 3 times in blotting solution, once in 0.5% (*v*/*v*) Triton X100 in PBS pH 7.4, and 2 times in 0.05 M Tris-HCl pH 6.8; the immunoreaction was performed using an ECL chemiluminescence detection system (Cytiva, Uppsala, Sweden), and signals were detected by exposing membranes to Hyperfilm ECL X-ray films (Cytiva, Uppsala, Sweden). WB images were analyzed using Melanie 9 software, using the intensity values of detected bands. Immunoblot data was then exported into Excel to perform statistical analysis by XLStat, version 2021.2.2, by Kruskal–Wallis two-tailed test and the post hoc Dunn’s test for multiple comparison. Protein bands were normalized on ACTB as housekeeping.

## 3. Results

### 3.1. Population

Patients enrolled in the study, of any ethnicity with a first diagnosis of urothelial carcinoma of the bladder suspected based on ultrasound, urine cytology, and cystoscopy, and histologically confirmed after endoscopic resection of the lesion (TURB), have a mean age of 71.4 ± 11 years for pTa-LG and 76.3 ± 7 years for pT1-HG. Selected patients have a mean weight of 76.5 ± 11 kg, corresponding to a Body Mass Index (BMI) of 26.2 ± 3 for pTa-LG and 78.6 ± 13 kg and a BMI of 27 ± 3 for pT1-HG. Forty percent of pTa-LG patients, against 46.10% of pT1-HGs, suffer from diabetes. Fifty percent of pTa-LG and 46.10% of pT1-HG are smokers and present hematuria. Tumor size of pTa-LG patients has a mean of 1.6 ± 0.4 cm and 2.4 ± 1.7 cm in pT1-HG (Table 1).

### 3.2. Proteomic Results

**Figure 2:**
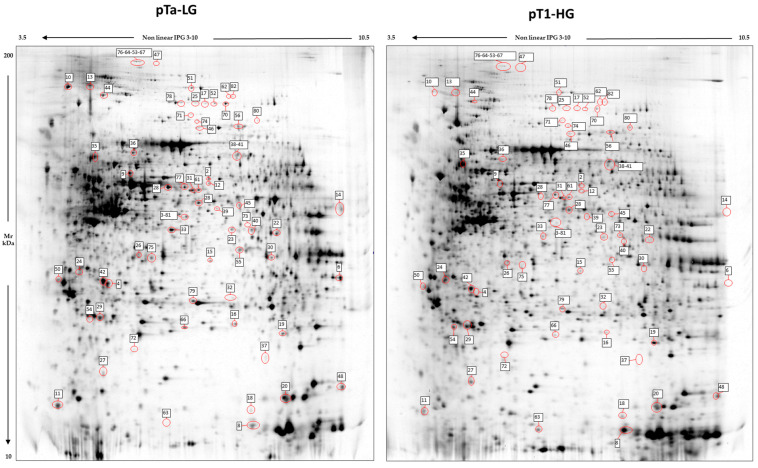
2D electrophoresis maps representative of pTa-LG and pT1-HG NMIBC biopsies, respectively. Maps reported a mean of 2800 protein spots, of which 68 were differentially abundant in pT1-HG with respect to pTa-LG biopsies. Spot numbers correspond to those reported in Table 2.

Image analysis by Melanie 9 software of the 2D-gel images obtained by 4 pTa-LG and 4 pT1-HG NMIBC biopsies from patients at first diagnosis of urothelial carcinoma of the bladder, was performed on 2800 spots present in all 2D gels. After comparison of the 2D images, 68 protein spots respected the parameters selected for the statistical analysis and were considered differentially abundant between pTa-LG and pT1-HG, and are reported in Figure 2. After MALDI-ToF MS analysis, we identified 60 proteins whose data are shown in Table 2.

**Figure 3:**
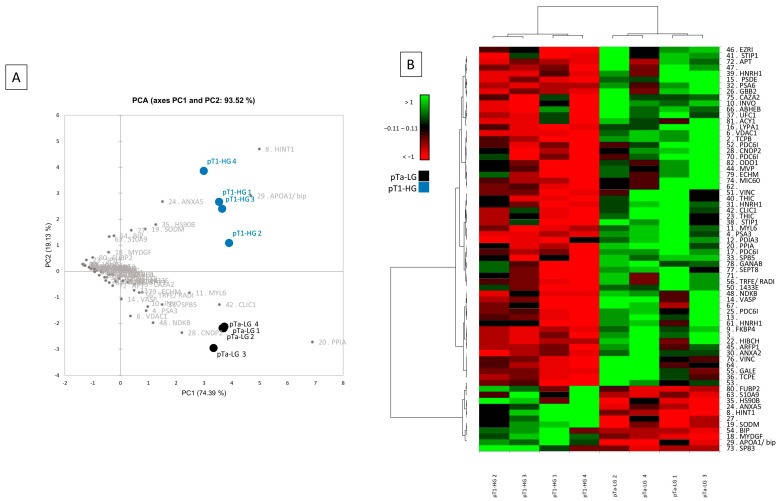
Multivariate analysis by (**A**) PCA and (**B**) heatmap analysis performed on the %V of the differentially abundant spots.

Multivariate statistical analysis by principal component analysis (PCA) and heatmap analysis were performed with a reliance on the %V of the 68 differentially abundant spots, and they are shown in Figure 3. PCA summarizes the 93.52% (PC1: 74.39% and PC2: 19.13%) of the variance highlighted as pTa-LG and pT1-HG samples clustered into two distinct groups along the PC2 (Figure 3A). Several differentially abundant proteins contributed significantly to the distribution of samples on the PCA plot, including HINT1, APOA1/BIP, ANXA5, HSP90B, SOD2, S10A9, MYDGF, PPIA, CLIC1, CNDP2, MYL6, SBP5, NDKB, VDAC1, PSA3, and INVO. Figure 3B shows a heatmap analysis highlighting that, significantly, samples from the same condition cluster together. Moreover, in the pT1-HG condition, a predominant downregulation of protein spots is observed.

### 3.3. Enrichment Analysis of the Highly Abundant Proteins in pT1-HG NMIBC

**Figure 4:**
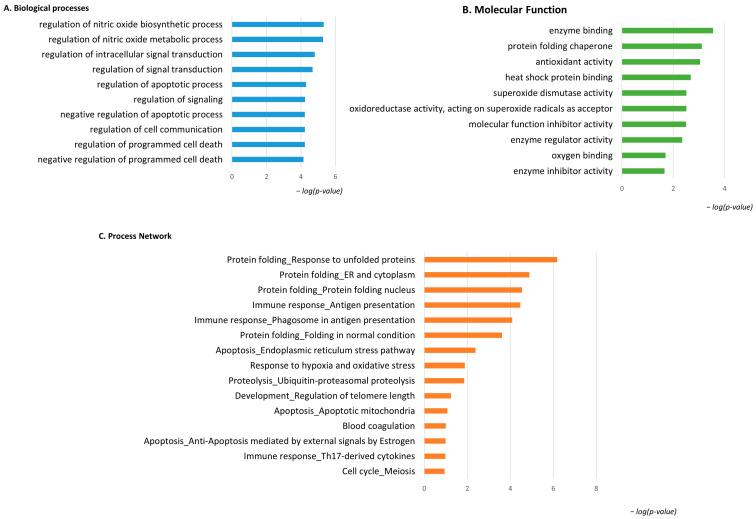
The Figure reports the biological processes (**A**) and molecular functions (**B**), revealed by the DAVID tool, of the highly abundant proteins in pT1-HG NMIBC. The Figure also reports the process network report (**C**).

Considering the spot behavior, the enrichment analysis that was performed on the two subgroups of differential proteins relies on their high or low abundance in pT1-HG NMIBC biopsies. Figure 4 shows the enrichment analysis performed by the DAVID functional annotation tool and by the MetaCore software on highly abundant proteins in pT1-HG biopsies. Biological processes (BP) by gene ontology (GO) (Figure 4A) highlighted regulation of the nitric oxide biosynthetic process, regulation of signal transduction, negative regulation of the apoptotic process, regulation of cell communication, and negative regulation of programmed cell death as the most enriched terms. Molecular function (MF) analysis (Figure 4B) of these proteins showed their activities as the following: enzyme binding, protein folding chaperone, antioxidant, heat shock protein binding, superoxide dismutase, molecular function inhibitory activity, enzyme regulator activity, oxygen binding, and enzyme inhibitory activity. Moreover, the enrichment analysis by the MetaCore software reported the process networks in Figure 4C and Table S2. Highly abundant proteins in pT1-HG cooperate in protein folding, in immune response, in regulation of apoptosis, in proteolysis, and in response to hypoxia and oxidative stress.

**Figure 5:**
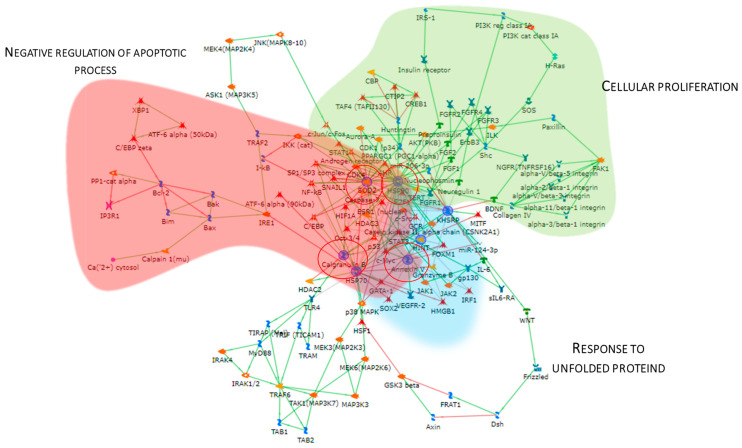
The Figure reports the protein network performed by the up-regulated proteins in pT1-HG, using the MetaCore software. Red circles highlight central hub proteins. Symbols meaning is reported into Supplementary materials as MetaCore legend.

The Metacore software also constructed the protein network (Figure 5), showing that the proteins were interconnected and indicating a close relationship. SOD2, HSP90, APOA1, KHSRP, and Annexin V (ANXA5) (red circles) were central functional hubs, i.e, proteins with a higher number of edges, suggesting their pivotal role in modulating more molecular pathways. We validate SOD2 and ANXA5 by Western blot, confirming their high abundance also in other pT1-HG biopsies (Figure 9). Red, green, and light blue clouds highlight parts of the protein net involved in the negative regulation of the apoptotic process, cellular proliferation, and response to unfolded proteins, respectively.

### 3.4. Enrichment Analysis of the Low-Abundant Proteins in pT1-HG NMIBC

**Figure 6:**
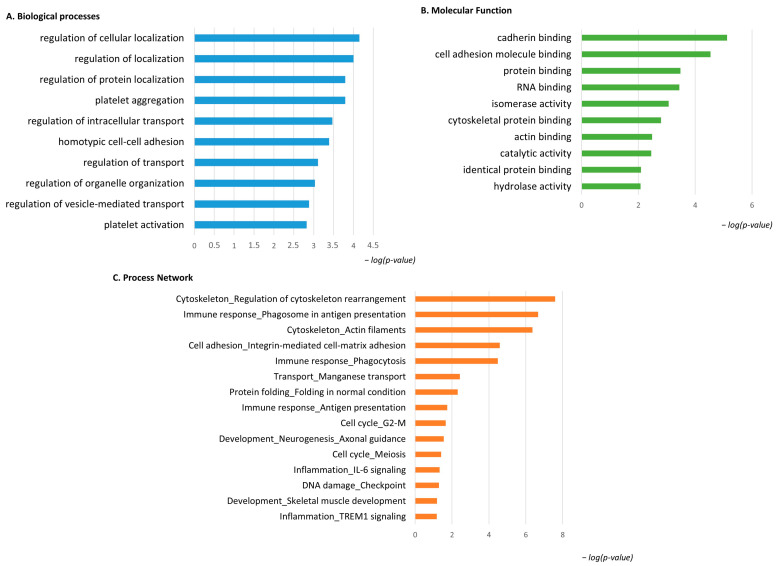
The Figure reports the biological processes (**A**) and molecular functions (**B**), revealed by the DAVID tool, of the low-abundant proteins in pT1-HG NMIBC. The Figure also reports the process network report (**C**).

The same enrichment analysis was performed for the low-abundant proteins in pT1-HG NMIBC. Figure 6A shows that the GO BP was enriched for the identified proteins. Regulation of cellular localization, regulation of protein localization, platelet aggregation and activation, regulation of intracellular transport and organelle organization, and regulation of vesicle-mediated transport were the most enriched terms for the low-abundant proteins in pT1-HG. Figure 6B reports MF related to cadherin binding, cell adhesion molecule binding, protein binding, RNA binding, isomerase activity, cytoskeletal protein binding, actin binding, catalytic activity, identical protein binding, and hydrolase activity. Process network performed by MetaCore is show into Figure 6C and Table S3 as evidence of how low-abundant proteins in pT1-HG NMIBC are involved in the regulation of cytoskeleton rearrangement, in immune response by antigen presentation and phagocytosis, in integrin mediated cell–matrix adhesion, in manganese transport, in protein folding, in the cell cycle, in neurogenesis and skeletal muscle development, in inflammation by IL6 signaling and TREM1 signaling, and in DNA damage.

**Figure 7:**
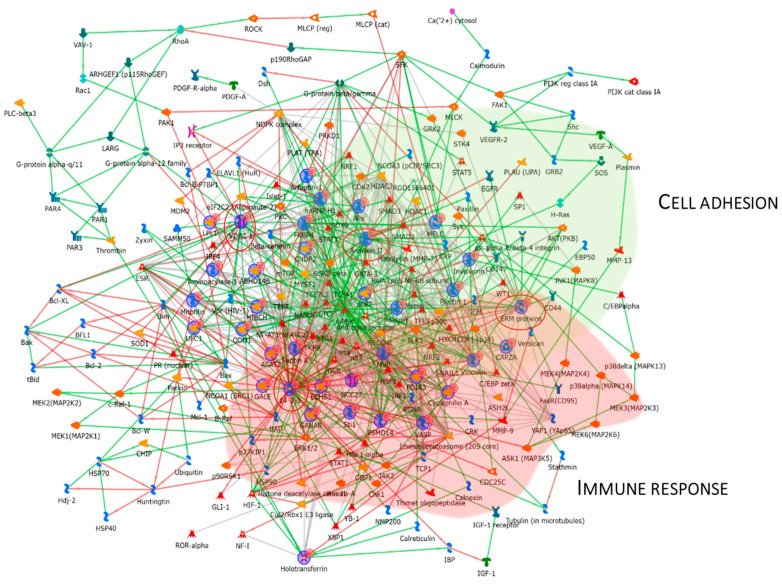
The Figure reports that the protein network analysis was performed based on downregulated proteins in pT1-HG, using MetaCore software. Red circles highlight central hub proteins. Symbols meaning is reported into Supplementary materials as MetaCore legend.

Figure 7 reports the protein network, and Annexin II, ERM proteins (ezrin), VDAC 1, and 14-3-3ε were considered central functional hubs. Western blot of 14-3-3ε also validates the low abundance of this protein in other pT1-HG biopsies with respect to pTa-LGs (Figure 9). Red and green clouds highlight parts of the protein net involved in immune response and cell adhesion, respectively.

### 3.5. Protein Abundance Correlations with Tumor Size and Tumor Stage

**Figure 8:**
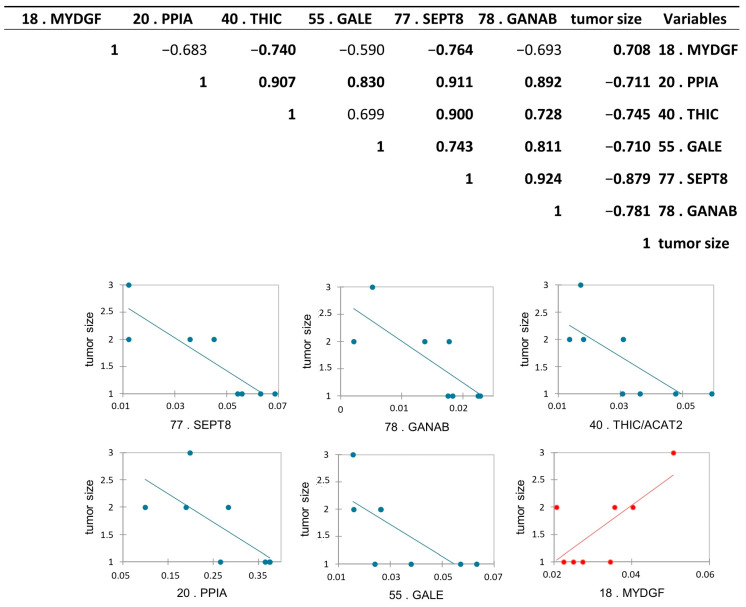
Pearson’s correlation analysis between differentially abundant proteoforms between pTa-LG and pT1-HG NMIBC biopsies and tumor size. Values in bold are different from 0, with a significance level alpha = 0.05. Positively correlated protein is reported in red, while the inversed correlated proteins to the tumor size were reported in blue.

Protein abundance correlation with tumor stage and with the tumor size obtained after TURB, and after histological analysis, was performed to find a possible relationship between the abundance of the proteins with the increase and the “invasiveness” of the cancer. To this purpose, we performed a Pearson’s correlation analysis among all differential proteins and tumor stages, and, as expected, all proteins were statistically correlated with the tumor stage, according to the proteomic results. Moreover, we performed the same analysis among differential proteins and tumor size. Interestingly, we found six differentially abundant proteoforms to be statistically significant: Peptidyl-prolyl cis-trans isomerase A (PPIA), UDP-glucose 4-epimerase (GALE), septin-8 (SEPT8), Acetyl-CoA acetyltransferase, cytosolic (THIC/ACAT2), and Neutral alpha-glucosidase AB (GANAB) were inversely correlated, while Myeloid-derived growth factor (MYDGF) was positively correlated with the tumor size with a *p*-value < 0.05 (Figure 8).

### 3.6. Protein Abundance Validation by Western Blot Analysis

**Figure 9:**
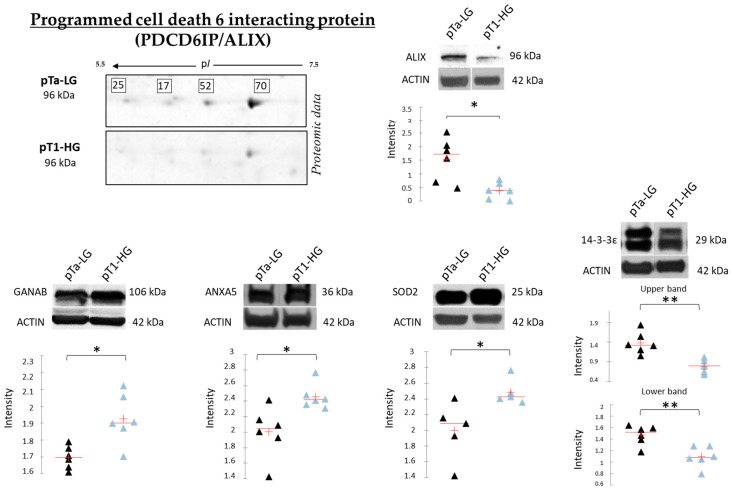
The Figure reports on the left part, on the upper side, the highlight of the 4 proteoforms of PDCD6IP/ALIX, all found downregulated in pT1-HG with respect to pTa-LG in top-down proteomic analysis. The Figure also reports mono-dimensional Western blot analysis of PDCD6IP/ALIX, GANAB, ANXA5, SOD2, and 14-3-3ε. Kruskal–Wallis two-tailed test and the post hoc Dunn’s test for multiple comparison were performed; * *p*-value < 0.05, ** *p*-value < 0.01. Black triangles represent pTa-LG samples while light blue triangles the pT1-HG ones.

Figure 9 reports the Western blot (WB) analyses performed to validate SOD2, ANXA5, 14-3-3ε, and GANAB, given their potential role in distinguishing pTa-LG and pT1-HG biopsies at the molecular level. Unlike the top-down proteomic approach, which highlights differences in proteoform abundance, one-dimensional WB analysis reflects variations in the total protein amount. In this context, we validated the proteomic results at the level of total protein for SOD2 and ANXA5, both of which were found to be more abundant in pT1-HG biopsies compared to pTa-LG. We also confirmed the downregulation of 14-3-3ε in pT1-HG samples, supporting the relevance of this protein in the dysregulation of downstream molecular pathways. WB analysis of GANAB revealed a higher total protein level in pT1-HG compared to pTa-LG, whereas the specific proteoform identified by proteomics (spot 78) displayed the opposite trend. Furthermore, the top-down proteomic approach identified four proteoforms of programmed cell death 6-interacting protein (PDCD6IP/ALIX), all of which were less abundant in pT1-HG relative to pTa-LG (2-DE image highlighted in Figure 9). Considering the well-documented association between ALIX function and cancer progression reported in the literature [20], we also validated this protein by WB. The results in Figure 9 strongly confirm its downregulation in the more aggressive NMIBC subtype, pT1-HG.

## 4. Discussion

Non-muscle-invasive bladder cancer (NMIBC) represents a clinically heterogeneous group of tumors characterized by highly variable risks of recurrence and progression, which depend on multiple clinical and pathological factors. According to the TNM classification, NMIBC includes mucosal tumors (pTa and pTis) and those invading the lamina propria (pT1). Although the long-term survival of patients with NMIBC is generally favorable—approximately 70–85% at 10 years for pTa disease—high-grade pT1 lesions are associated with a substantial risk of recurrence (approximately 45%) and a notable likelihood of progression (around 17%). Therefore, accurately predicting the risk of recurrence and progression in NMIBC based on individual patient and disease characteristics holds considerable prognostic value. Effective risk stratification in NMIBC can enable personalized treatment strategies and tailored surveillance protocols, representing a significant step away from the traditional “one-size-fits-all” approach [21].

Proteomic analysis is a powerful approach for gaining unique insights into disease biology. In this study, a top-down proteomic strategy was employed to identify differential proteoforms between the most distinct subclasses of NMIBC disease severity—pTa-LG and pT1-HG—in order to elucidate the molecular mechanisms underlying the potential transition toward the more aggressive form. Our analysis revealed that, among the dysregulated proteins identified, the majority were downregulated in the more severe subtype (pT1-HG) compared to the less severe one (pTa-LG). Proteins that decreased in abundance with increasing tumor severity were mainly associated with cytoskeleton remodeling, intracellular transport, and cell–cell adhesion (ARFIP1, ANXA2, CCT2, CCT5, EZR, LYPLA1, PPIA, RDX, SEPTIN8, 14-3-3ε, and VCL)—mechanisms well known to contribute to tumor growth, tissue invasion, and subsequent detachment of cancer cells for metastasis [22]. Among these, 14-3-3ε, we found a highly conserved regulatory protein that interacts with hundreds of structurally diverse clients and acts as a central hub of signaling networks, such as cell-cycle control, apoptosis, cell adhesion, and localization of signaling molecules, downregulated in pT1-HG [23,24]. To the best of our knowledge, there are no studies on the involvement of 14-3-3ε in bladder cancer. However, a recent review highlighted that 14-3-3ε is involved in tumor invasion, migration, and metastasis in many cancer types [24]. The decrease in this protein could be validated as a prognostic biomarker in NMIBC. Interestingly, ezrin and radixin, which we found downregulated in pT1-HG, belong to the Ezrin/Radixin Moesin complex (ERM), a scaffold protein that crosslinks plasma membrane proteins with the actin cytoskeleton [25]. In addition to their role in cytoskeleton regulation and in providing a platform for the transmission of signals in response to extracellular cues [26], they are also involved in mediating plasma membrane localization of programmed cell death 1 ligand 1 (PD-L1), possibly by post-translational modifications without affecting PD-L1 transcription [25,27]. Taken together with our findings, these results suggest that targeting EZR and RDX may represent a novel therapeutic strategy to enhance the efficacy of PD-1/PD-L1 blockade therapies. CCT2 and CCT5 are two additional proteins found to decrease in abundance with increasing tumor severity. Both are subunits of the chaperonin-containing TCP-1 (CCT or TRiC) complex, a multi-subunit molecular chaperone responsible for the folding of approximately 10% of cytosolic protein substrates, including cytoskeletal components, cell-cycle regulators, and aggregation-prone proteins [28].

Of relevance, among the downregulated proteins identified in pT1-HG, we detected four proteoforms of PDCD6IP (also known as ALIX), an accessory component of the Endosomal Sorting Complexes Required for Transport (ESCRT) machinery. Notably, ALIX has been reported to regulate both epidermal growth factor receptor (EGFR) activity and PD-L1 surface presentation in basal-like breast cancer (BLBC) cells. Loss of ALIX results in prolonged and enhanced stimulation-induced EGFR activation, as well as defective PD-L1 trafficking through multivesicular bodies, leading to reduced exosomal secretion and redistribution of PD-L1 to the plasma membrane. Moreover, mouse models of breast cancer have shown that ALIX-deficient tumors are larger and exhibit a more immunosuppressive tumor microenvironment [20]. Western blot analysis of independent samples confirmed the reduced abundance of PDCD6IP/ALIX in pT1-HG compared to pTa-LG biopsies, highlighting ALIX as a promising target for future studies aimed at improving NMIBC stratification.

On the other hand, the up-regulated proteins in pT1-HG belong to biological processes known to be involved in cancer progression, such as the regulation of nitric oxide (NO) biosynthetic process (KHSRP, SOD2, and HSP90), regulation of signal transduction (S100A9, SOD2, APOA1, HSP90, HSPA5, HINT1, MYDGF, and SerpinB3), and in negative regulation of apoptosis (SOD2, ANXA5, HSP90B1, HSPA5, and MYDGF). Consistent with our findings, inducible nitric oxide synthase (iNOS) is expressed in 50% of human bladder cancers and is associated with poor prognosis. Although Bacillus Calmette–Guerin (BCG) is the standard treatment for high-risk NMIBC, 30–50% of patients show resistance, which correlates positively with iNOS expression. S100A9, which we found up-regulated in pT1-HG biopsies, was identified as a protein associated with iNOS activity [29], and it is also involved in the regulation of signal transduction, such as SOD2, APOA1, HSP90, HSPA5, HINT1, MYDGF, and SerpinB3, and in immunomodulation in tumor microenvironments. Also, SOD2 has an important role in tumor development and response to treatment. In accordance with our results, which were also validated by WB in a wider sample population, Singh et al. reported a significant correlation between oxidative stress markers and NMIBC pT1-HG [30]. Up-regulated proteins in pT1-HG are also involved in the negative regulation of the apoptotic process. Among these proteins, there are SOD2, ANXA5, HSP90B1, HSPA5, and MYDGF. Several studies have reported that high ANXA5 levels, as observed in the most severe form, are associated with significantly lower survival rates in various cancers, highlighting its critical role in proliferation, metastasis, and immune evasion [31,32]. Up-regulated proteins in pT1-HG biopsies are also involved in protein folding (HSP90, HSP70, and HSPA5), oxidative stress (SOD2, HSP90), and immune response (HSP90, HSP70, and HSPA5), three fundamental molecular processes that malignant cells utilize to enable them to thrive under adverse conditions while simultaneously inhibiting the development of anti-tumor immune responses [33]. Moreover, cancer cells have a higher dependency on molecular chaperones, such as HSP90, since they are uniquely challenged due to imbalances caused by chromosomal abnormalities and overexpression of oncogenes, ultimately leading to cellular stress [34]. Interestingly, it was reported that inhibitors of HSP90 showed promising outcomes in the treatment of metastatic breast cancer [35]. Furthermore, it is important to consider that these proteins belong, for a major part, to extracellular exosome/extracellular membrane-bounded organelles, suggesting that they can be secreted by the cancerogenic cells and could be released in biological fluids such as urine and serum, where they can be revealed as prognostic biomarkers.

### 4.1. Protein Correlation with Tumor Size

To investigate the prognostic potential of specific proteins, we performed a correlation analysis between the differentially abundant proteins and tumor stage and size. While all proteins, as expected, were correlated with tumor stage according to proteomic data, GANAB, THIC/ACAT2, SEPT8, PPIA, and GALE showed an inverse correlation with tumor size, whereas MYDGF displayed a positive correlation.

Of particular interest, GANAB and GALE are both enzymes critically involved in glycoprotein and glycolipid synthesis in normal and neoplastic cells [36,37,38]. Protein and lipid glycosylation is a common post- and co-translational modification involved in numerous cellular processes and frequently altered in cancer, affecting tumor angiogenesis, invasion, and metastasis [39,40]. Western blot analysis of GANAB revealed an upregulation of the total protein in pT1-HG, contrasting with the downregulation of the proteoform identified in spot 78. As reported in the UniProt database, GANAB is a protein that can undergo modifications to numerous amino acid residues, modulating its function and interactions. Several studies [41,42] have reported that GANAB can be glycosylated and modified by hydroxyproline (Pro223). This suggests that the proteoform we found to be downregulated in pT1-HG using a 2D gel-based proteomic approach is characterized by a specific post-translational modification (PTM) that confers a distinct functional activity. In turn, this activity may become impaired during disease progression and making the investigation of PTMs on this protein of particular interest. Nonetheless, the dysregulation of the amount of both GANAB and GALE suggests that alterations in their expression may lead to aberrant glycosylation, thereby contributing to tumor progression in NMIBC.

THIC/ACAT2, another protein inversely correlated with tumor size, is involved in the cholesterol biosynthesis pathway. Dysregulation of ACAT2 has been associated with various cancers [43,44,45,46]. Septin-8 (SEPT8), also inversely correlated with tumor size, has previously been reported in “The Human Protein Atlas” database as a favorable prognostic marker in bladder urothelial carcinoma. Beyond its role in cytokinesis, SEPT8 participates in vesicle trafficking [47], exocytosis [48], apoptosis [49], and tumorigenesis [50]. On the other hand, MYDGF/C19orf10 was positively correlated with tumor size. MYDGF is a novel secreted protein with potent antiapoptotic and tissue-repairing properties. The role of MYDGF in health and disease may involve cell apoptosis and proliferation, tissue repair and regeneration, anti-inflammation, and glycolipid metabolism regulation [51], according to GANAB and GALE activities. Interestingly, MYDGF/C19orf10 is a protein promoting proliferation, migration, and invasion of bladder cancer cells, thereby contributing to the progression and malignant behavior of the disease by influencing pathways like PI3K/AKT and Wnt/β-catenin [51,52], as also suggested by our protein network.

### 4.2. Strengths and Limitations

The main strength of this study lies in the use of fresh biopsies obtained after TURB for comprehensive proteomic analysis, allowing us to examine protein dynamics during tumor progression from pTa-LG to pT1-HG in terms of histological grade and invasiveness. This approach enabled the identification of key modulated molecular mechanisms and highlighted proteins with pivotal roles across different NMIBC stages, which were further validated by Western blot analysis on independent samples. However, the study is limited by the small sample size and small number of validated proteins, in addition to the use of one-dimensional Western blotting for protein validation, which does not provide information on proteoforms. MALDI-TOF mass spectrometry was chosen for protein identification because of its ability to rapidly and sensitively identify proteins at femtomole levels. Indeed, this approach allows the identification of up to 96 protein samples simultaneously, which is particularly advantageous when a two-dimensional gel electrophoresis method is used to resolve protein samples. Additionally, while our findings implicate several pathways, the precise mechanistic details remain to be elucidated. Future studies will aim to increase the sample size, include additional disease stages, and apply alternative proteomic techniques, such as shotgun proteomics, to deepen biomarker stratification research in NMIBC.

## 5. Conclusions

In this study, a top-down proteomic approach revealed significant differences in proteoform profiles between low-grade (pTa-LG) and high-grade (pT1-HG) NMIBC, highlighting that most proteoforms were downregulated in the more aggressive subtype (pT1-HG). Among these, 14-3-3ε—implicated in tumor invasiveness and metastasis—and PDCD6IP/ALIX—a key regulator of EGFR activity and immune checkpoint trafficking—were also validated by Western blot analysis. Conversely, proteins up-regulated in pT1-HG included SOD2 and ANXA5, which were both strongly validated by Western blot. ANXA5, which is normally expressed at low levels in the urinary bladder (The Human Protein Atlas), represents an interesting biomarker deserving further exploration. Moreover, both ANXA5 and SOD2, as central hubs of the protein network, reflect their pivotal roles in numerous downstream molecular pathways. Interestingly, proteins inversely correlated with tumor size included GANAB and GALE. In particular, the proteoform of GANAB found to be downregulated during disease progression is worthy of further investigation regarding its PTMs.

Overall, these findings provide novel insights into the molecular mechanisms underlying NMIBC progression and identify promising biomarkers—such as 14-3-3ε, ALIX, SOD2, ANXA5, and GANAB—for future prognostic evaluation and therapeutic targeting.

## Figures and Tables

**Table 1:** Patients’ information.

	pTa-LG	pT1-HG
AGE	71.4 ± 11	76.3 ± 7
WEIGHT (Kg)	76.5 ± 11	78.6 ± 13
BMI	26.2 ± 3	27 ± 3
DIABETES %	40	46.10
SMOKER %	50	46.10
HYPERTENSION %	50	46.10
HAEMATURIA %	70	92
TUMOR SIZE (cm)	1.6 ± 0.4	2.4 ± 1.7

**Table 2:** Table of differentially abundant proteins by proteomic analysis.

Spot N.	Protein Name	Entry Name _HUMAN	Gene Name	AC	Anova Test				Experimental pI-MW (kDa)	Theorical pI-MW (Da)	Mascot Search Results		
					Anova (*p*)	pT1-HG	pTa-LG	FC			Score	No. of Matched Peptides	Sequence Coverage (%)
2	T-complex protein 1 subunit beta	TCPB	*CCT2*	P78371	4.62 × 10^−4^	0.008	0.0226	2.932	5.95–46	6.01–57,794	148	12/17	33
36	T-complex protein 1 subunit epsilon	TCPE	*CCT5*	P48643	0.0171	0.0121	0.0421	3.475	5.33–55	5.45–60,089	121	9/11	24
4	Proteasome subunit alpha type-3	PSA3	*PSMA3*	P25788	5.98 × 10^−4^	0.0263	0.1294	4.932	5.05–27	5.19–28,643	174	12/16	37
32	Proteasome subunit alpha type-6	PSA6	*PSMA6*	P60900	0.01541	0.015	0.0305	2.032	6.15–26	6.34–27,838	87	6/11	25
15	26S proteasome non-ATPase regulatory subunit 14	PSDE	*PSMD14*	O00487	0.0048	0.0152	0.0303	1.997	5.94–31	6.06–34,726	151	12/16	44
6	Non-selective voltage-gated ion channel VDAC1	VDAC1	*VDAC1*	P21796	7.43 × 10^−4^	0.00868	0.1096	12.627	9.53–29	8.62–30,868	228	13/16	61
8	Adenosine 5′-monophosphoramidase HINT1	HINT1	*HINT1*	P49773	0.0027	0.2921	0.1240	2.354	6.46–11	6.43–13,907	75	4/8	51
9	Peptidyl-prolyl cis-trans isomerase FKBP4	FKBP4	*FKBP4*	Q02790	0.0034	0.0302	0.0682	2.261	5.28–47	5.35–52,057	217	17/21	32
10	Involucrin	INVO	*IVL*	P07476	0.0035	0.0366	0.1212	3.311	4.45–109	4.62–68,551	261	19/25	42
11	Myosin light polypeptide 6	MYL6	*MYL6*	P60660	0.0037	0.0916	0.164	1.791	4.3–13	4.56–16,930			
12	Protein disulfide-isomerase A3	PDIA3	*PDIA3*	P30101	0.0041	0.0092	0.0179	1.955	5.94–45	5.98–57,146	111	13/24	23
14	Vasodilator-stimulated phosphoprotein	VASP	*VASP*	P50552	0.0047	0.0129	0.0812	6.3094	9.86–40	9.05–39,976	74	6/14	21
16	Acyl-protein thioesterase 1	LYPA1	*LYPLA1*	O75608	0.00502	0.0165	0.0443	2.692	6.23–22	6.29–24,996	89	5/8	37
17	Programmed cell death 6-interacting protein	PDC6I	*PDCD6IP*	Q8WUM4	0.00515	0.0104	0.024	2.3103	5.95–89	6.13–96,590	60	5/7	8
25	Programmed cell death 6-interacting protein	PDC6I	*PDCD6IP*	Q8WUM4	0.01114	0.0058	0.014	2.411	5.85–90	6.13–96,590	67	6/5	8
52	Programmed cell death 6-interacting protein	PDC6I	*PDCD6IP*	Q8WUM4	0.0263	0.0082	0.0237	2.878	6.02–89	6.13–96,590	160	12/14	21
70	Programmed cell death 6-interacting protein	PDC6I	*PDCD6IP*	Q8WUM4	0.0366	0.02442	0.0564	2.310	6.11–90	6.13–96,590	304	22/23	34
18	Myeloid-derived growth factor	MYDGF	*MYDGF*	Q969H8	0.0063	0.0402	0.0238	1.687	6.38–13	6.20–18,897	69	5/8	27
19	Superoxide dismutase [Mn], mitochondrial	SODM	*SOD2*	P04179	0.0064	0.099	0.052	1.916	7.12–21	8.35–24,906	118	7/11	41
20	Peptidyl-prolyl cis-trans isomerase A	PPIA	*PPIA*	P62937	0.0075	0.188	0.3493	1.857	7.34–15	7.68–18,229	222	13/17	71
22	3-hydroxyisobutyryl-CoA hydrolase, mitochondrial	HIBCH	*HIBCH*	Q6NVY1	0.0106	0.0113	0.0467	4.11	7.12–34	8.38–43,797	64	5/8	14
23	Acetyl-CoA acetyltransferase, cytosolic	THIC	*ACAT2*	Q9BWD1	0.0106	0.0201	0.0366	1.822	6.2–36	6.47–41,838	102	7/11	23
40	Acetyl-CoA acetyltransferase, cytosolic	THIC	*ACAT2*	Q9BWD1	0.0178	0.0198	0.0436	2.197	6.48–36	6.47–41,838	138	8/9	29
24	Annexin A5	ANXA5	*ANXA5*	P08758	0.011	0.141	0.051	2.767	4.66–28	4.94–35,971	173	12/20	41
30	Annexin A2	ANXA2	*ANXA2*	P07355	0.01393	0.0108	0.0201	1.857	6.99–31	7.57–38,808	113	8/12	32
26	Guanine nucleotide-binding protein G(I)/G(S)/G(T) subunit beta-2	GBB2	*GNB2*	P62879	0.01126	0.0097	0.0335	3.449	5.37–31	5.60–38,048	87	8/17	21
28	Cytosolic non-specific dipeptidase	CNDP2	*CNDP2*	Q96KP4	0.0123	0.0565	0.1804	3.195	5.61–45	5.66–53,187	125	9/13	24
29	**MIX**										236		
	Apolipoprotein A-I	APOA1	*APOA1*	P02647	0.0123	0.2501	0.1462	1.711	4.98–22	5.56–30,759	183	19/47	49
	Endoplasmic reticulum chaperone BiP Fragment C	BIP	*HSPA5*	P11021						5.07–72,402	80	13/47	19
54	Endoplasmic reticulum chaperone BiP Fragment C	BIP	*HSPA5*	P11021	0.0276	0.063	0.012	5.3	4.79–22	5.07–72,402	78	7/14	13
35	Heat shock protein HSP 90-beta	HS90B	*HSP90AB1*	P08238	0.0168	0.115	0.056	2.059	4.89–55	4.97–83,554	156	18/27	23
31	Heterogeneous nuclear ribonucleoprotein H	HNRH1	*HNRNPH1*	P31943	0.0153	0.024	0.0481	2.032	5.8–43	5.89–49,484	197	15/24	48
39	Heterogeneous nuclear ribonucleoprotein H	HNRH1	*HNRNPH1*	P31943	0.0177	0.0109	0.0222	2.034	6.03–39	5.89–49,484	75	6/13	22
61	Heterogeneous nuclear ribonucleoprotein H	HNRH1	*HNRNPH1*	P31943	0.03219	0.0151	0.032	2.089	5.85–43	5.89–49,484	124	9/13	28
73	Serpin B3	SPB3	*SERPINB3*	P29508	0.0383	0.0088	0.0024	3.758	6.41–37	6.35–44,594	220	16/18	42
33	Serpin B5	SPB5	*SERPINB5*	P36952	0.0155	0.057	0.132	2.325	5.62–35	5.72–42,530	223	15/21	51
37	Ubiquitin-fold modifier-conjugating enzyme 1	UFC1	*UFC1*	Q9Y3C8	0.0172	0.01	0.02	2.024	6.65–19	6.90–19,617	75	5/9	28
38	Stress-induced-phosphoprotein 1	STIP1	*STIP1*	P31948	0.0172	0.034	0.0754	2.221	6.25–55	6.40–63,227	183	14/14	25
41	Stress-induced-phosphoprotein 1	STIP1	*STIP1*	P31948	0.0178	0.011	0.0263	2.5185	6.26–53	6.40–63,227	137	13/17	24
42	Chloride intracellular channel protein 1	CLIC1	*CLIC1*	O00299	0.01789	0.115	0.208	1.81028	5.01–27	5.09–27,248	120	7/10	37
44	Major vault protein	MVP	*MVP*	Q14764	0.0207	0.0058	0.0166	2.83339	5.03–98	5.34–99,551	387	27/28	40
45	Arfaptin-1	ARFP1	*ARFIP1*	P53367	0.0212	0.0104	0.0217	2.07471	6.28–40	6.24–41,770	141	9/12	35
48	Nucleoside diphosphate kinase B	NDKB	*NME2*	P22392	0.0246	0.0282	0.1414	5.02072	9.24–16	8.52–17,401	130	9/14	59
50	14-3-3 protein epsilon	1433E	*YWHAE*	P62258	0.0256	0.040	0.073	1.8259	4.26–27	4.63–29,326	221	17/20	47
51	Vinculin	VINC	*VCL*	P18206	0.0261	0.006	0.018	3.16065	5.78–106	5.50–124,292	130	13/16	15
55	UDP-glucose 4-epimerase	GALE	*GALE*	Q14376	0.028	0.0204	0.046	2.26711	6.27–32	6.26–38,656			
78	Neutral alpha-glucosidase AB	GANAB	*GANAB*	Q14697	0.0427	0.01	0.0202	2.045	5.73–89	5.74–107,263	239	2/25	25
46	Ezrin	EZRI	*EZR*	P15311	0.022	0.0099	0.022	2.174	5.88–68	5.94–69,484	205	21/31	34
56	**MIX**										228		
	Serotransferrin	TRFE	*TF*	P02787	0.0285	0.039	0.0962	2.449	6.29–67	6.81–79,280	119	15/38	25
	Radixin	RADI	*RDX*	P35241						6.03–68,635	113	15/38	21
63	Protein S100-A9	S10A9	*S100A9*	P06702	0.0332	0.052	0.014	3.599	5.57–11	5.71–13,291			
64	Plectin *fragm*. (aa. 1061–1854)	PLEC	*PLEC*	Q15149	0.0334	0.0014	0.0059	4.239	5.32–142	5.74–533,462	91	13/13	3
66	Putative protein-lysine deacylase ABHD14B	ABHEB	*ABHD14B*	Q96IU4	0.0337	0.0251	0.051	2.012	5.72–22	5.94–22,446	154	8/9	45
72	Adenine phosphoribosyltransferase	APT	*APRT*	P07741	0.0382	0.014	0.061	4.2812	5.31–19	5.78–19,766	183	10/14	71
74	MICOS complex subunit MIC60	MIC60	*IMMT*	Q16891	0.038	0.005	0.0132	2.436	5.86–72	6.08–84,025	140	9/9	17
75	F-actin-capping protein subunit alpha-2	CAZA2	*CAPZA2*	P47755	0.03991	0.0418	0.0824	1.97	5.47–31	5.57–33,157	222	12/13	55
76	Vinculin	VINC	*VCL*	P18206	0.0405	0.001	0.004	3.853	5.3–142	5.50–124,292	253	23/26	27
77	Septin-8	SEPT8	*SEPTIN8*	Q92599	0.0413	0.029	0.058	2.035	5.73–44	5.89–56,234	194	15/19	30
79	Enoyl-CoA hydratase, mitochondrial	ECHM	*ECHS1*	P30084	0.0456	0.045	0.097	2.123	5.81–25	8.34–31,823	183	10/11	43
80	Far upstream element-binding protein 2	FUBP2	*KHSRP*	Q92945	0.0458	0.0173	0.01	1.827	6.58–73	6.85–73,355	265	18/20	35
81	Aminoacylase-1	ACY1	*ACY1*	Q03154	0.0459	0.016	0.052	3.339	5.74–38	5.77–46,084	161	10/12	32
82	2-oxoglutarate dehydrogenase complex component E1	ODO1	*OGDH*	Q02218	0.047	0.003	0.007	2.529	6.16–101	6.40–117,059	165	14/16	15

Table reports the spot numbers corresponding to those in Figure 2, the protein names of the identified spot by MALDI-ToF MS, UniProt Entry name and gene name, accession number (AC), the *p*-value ANOVA Test and the mean of the %V of the specific spot in pTa-LG and pT1-HG conditions, and the fold change in the %V means ratio between pTa-LG and pT1-HG, pI and MW. The last part of the Table is dedicated to Mascot Search Results, such as score, matched peptides, and sequence coverage (%). The proteins chosen for validation by Western blot are reported in red.

## Data Availability

The mass spectrometry proteomics data have been deposited to the ProteomeXchange Consortium via the PRIDE partner repository with the dataset identifier PXD069436.

## Supplementary Materials

The following supporting information can be downloaded at: https://www.mdpi.com/article/10.3390/proteomes13040065/s1, MetaCore legend; Table S1: Table reporting antibodies used for protein validation by WB; Table S2: Enrichment by Process Networks of up-regulated proteins in pT1-HG; Table S3. Enrichment by Process Networks of up-regulated proteins in pTa-LG.
