# Supplementary material for: Proteomic Profiling of Non-Muscle Invasive Bladder Cancer Reveals Stage-Specific Molecular Signatures and Prognostic Biomarkers
Source: Proteomes. 2025 Dec 10;13(4):65. doi: 10.3390/proteomes13040065 (PMC12736457; doi:10.3390/proteomes13040065)
Supplement: Supplementary file 1 [file proteomes-13-00065-s001.zip › MetaCore Legend.pdf]

Legend

MetaCore

Objects

Enzymes	
Generic Enzymes	
**Kinases**	**Phosphatases**
Generic Kinases	Generic phosphatases
Protein Kinases	Protein phosphatases
Lipid Kinases	Lipid phosphatases
**Phospholipases**	
Generic Phospholipases	
**Protease**	**Receptors**
Generic protease	Generic Receptors
Metalloproteases	G-Protein-Coupled Receptors (GPCR)
	Receptors with kinase activity

Generic objects
Receptor ligands
Transcription Factors
Proteins *General only*
Proteins *Binding*
Proteins *Membrane glycoprotein*
Compounds *MetaCore compound*
Compounds *Predicted metabolite or user's structure*
Inorganic ions
Reactions *Biochemical reaction, Transport reaction, Particle transformation, Protein processing, or RNA maturation*
DNA
RNA

© 2023 Clarivate. Clarivate and its logo, as well as all other trademarks used herein are trademarks of their respective owners and used under license.

Clarivate™

Membrane Transport Proteins	GTPases and adaptor/regulators	Groups of objects
Generic Channels	GTP-Binding Protein alpha Subunits (G-alpha)	A complex or a group<br>*Objects physically connected into a complex or related as a family, manually curated*
Ligand-Gated Ion Channels	Monomeric GTP-Binding Proteins (Small GTPases)	Related objects on networks<br>*Objects that are grouped automatically as part of network building. R-click to expand*
Voltage-Gated Ion Channels	GTP-Binding Protein beta/gamma Subunits (G beta/gamma)	Group 1<br>Custom Associations<br>*Group of objects created by user*
Transporters	GTP-Binding Protein Regulators (GDI, GAP, GEF, etc.)	

Objects on Maps

Cellular Structures	Other Objects on Maps
Mitochondria	Organism-specific object<br>*Object described only for a specific organism or group of organisms*
Endoplasmic Reticulum	Path Start
Golgi Apparatus	Text — Note
Cell Nucleus	Text — Normal Processes<br>*Static description*
Lysosomes	Normal Processes<br>*Links to metabolic and regulatory maps*
Peroxisomes	Text — Pathologic Processes
Cytoplasm	Pathological Processes<br>*Links to disease and tox process maps*
Extracellular Space	

Interactions

© 2023 Clarivate. Clarivate and its logo, as well as all other trademarks used herein are trademarks of their respective owners and used under license.

Interactions have attributes, direction, mechanism, and effect.

Direction

Interactions on Networks	Canonical Signal Transduction Pathways
Incoming Interactions<br>*Mouse-over an object to see incoming and outgoing interactions*<br><br>Outgoing Interactions	Canonical Pathways<br>*Pathways used in networks*<br><br>Canonical Pathways<br>*Pathways highlighted using Show/Hide*
**If you have uploaded your own Interactions using MetaLink**	**Interactions on Maps**
Interaction in network<br>*The interaction is present in both your uploaded dataset AND in MetaCore AND was included by the network building algorithm*<br><br>Interaction not in network<br>*The interaction is present in both your uploaded dataset AND in MetaCore BUT was excluded by the network building algorithm. It is only shown for information purposes*<br><br>Interaction not in MetaCore<br>*The interaction is present in your uploaded dataset BUT NOT in MetaCore*	Disrupted<br>*Interaction disrupted in disease*<br><br>Weakened<br>*Interaction weakened in disease*<br><br>Emergent<br>*Interaction emergent in disease*<br><br>Enhanced<br>*Interaction enhanced in disease*<br><br>Organism-specific interaction<br>*Interaction described only for a specific organism or group of organisms*

© 2023 Clarivate. Clarivate and its logo, as well as all other trademarks used herein are trademarks of their respective owners and used under license.

Mechanisms

Physical interactions

**B** Binding<br>*Physical interaction between two or more molecules*	**Tn** Biological Transport<br>*Transport of a molecule from one cellular compartment (including extracellular) to another*
**C** Cleavage<br>*Molecule is split into two or more fragments*	**Z** Catalysis<br>*Chemical reaction facilitated by an enzyme*
**CM** Covalent Modification<br>*Chemical modification of functional groups on proteins or nucleotides*	**Tr** Transcription Regulation<br>*Transcription factor binds to target gene's promoter to initiate/stimulate/terminate transcription*
**+P** Phosphorylation<br>*Addition of a phosphoryl group by a kinase*	**cRT** Co-regulation of Transcription<br>*Physical interaction of a transcription coregulator to target gene's promoter*
**-P** Dephosphorylation<br>*Removal of a phosphoryl group by a phosphatase*	**Rg** Regulation<br>*Influence on reaction, not defined by Catalysis mechanism*
**T** Transformation<br>*GTP/GDP exchange of a GTPase induced by regulators*	**M** MicroRNA Binding<br>*Physical interaction between a microRNA and its target mRNA*

Functional interactions

**IE** Influence on Expression<br>*Molecule has an indirect influence on gene expression*	**PE** Pharmacological Effect caused by drug interactions<br>*The action of a drug that affects the activity or metabolism of another drug*
**Cn** Competition<br>*Two or more molecules compete for binding to a target*	**TE** Toxic Effect caused by drug interactions<br>*The action of a drug that affects the toxicity of another drug*
**?** Unspecified<br>*Positive or negative effect on target molecule without defined mechanism*	

© 2023 Clarivate. Clarivate and its logo, as well as all other trademarks used herein are trademarks of their respective owners and used under license.

Clarivate™

Related objects	Effects
GR Group<br>*Relationship between objects considered as a unit*	Positive / activation
CS Complex-Subunit<br>*Relation between a complex and its subunits*	Negative / inhibition
SR Similarity<br>*Chemical compounds with a similar Tanimoto score*	Unspecified

Experiments

How your experimental data interacts with MetaCore data

On networks		On maps
	Up regulated object	
	Down-regulated object	
	Object with mixed signal<br>*Experiment contains both up- and down-regulated signals that map to a single object on the map/network*	
	Gene variants<br>*Variants in a VX file map to this object*	
	Multi-omics data<br>*Experimental data from multiple datatypes, such as gene expression and gene variant, map to the same object*	

For more information contact Customer Service at **LS Product Support.**

© 2023 Clarivate. Clarivate and its logo, as well as all other trademarks used herein are trademarks of their respective owners and used under license.
