# Supplementary material for: Proteomic Profiling of Non-Muscle Invasive Bladder Cancer Reveals Stage-Specific Molecular Signatures and Prognostic Biomarkers
Source: Proteomes. 2025 Dec 10;13(4):65. doi: 10.3390/proteomes13040065 (PMC12736457; doi:10.3390/proteomes13040065)
Supplement: Supplementary file 1 [file proteomes-13-00065-s001.zip › Table S1.pdf]

**Antibodies**	**Code**	**Dilution**	**Company**
Rabbit Anti-GANAB Polyclonal	PA589484	1:1000	ThermoFisher Scientific
Mouse Anti-Alix Monoclonal	MA183977	1:1000	ThermoFisher Scientific
mouse Anti-SOD2/MnSOD	ab16956	1:2000	Abcam
Mouse Anti-Annexin V	A8604	1:1000	Merk Sigma-Aldrich
Rabbit Anti-YWHAE	HPA008445	1:1000	Merk Sigma-Aldrich
Rabbit Anti-ß-Actin	4967	1:1000	Cell Signaling Technology
Mouse Anti-β-Actin	A2228	1:40000	Merk Sigma-Aldrich
goat peroxidase-conjugated anti-rabbit immunoglobulin G	A0545-1ML	1:7000	Merk Sigma-Aldrich
goat peroxidase-conjugated anti-mouse immunoglobulin G	A2554	1:3000	Merk Sigma-Aldrich

Table S1. Table reports the antibodies used for protein validation by monodimensional western blot.
